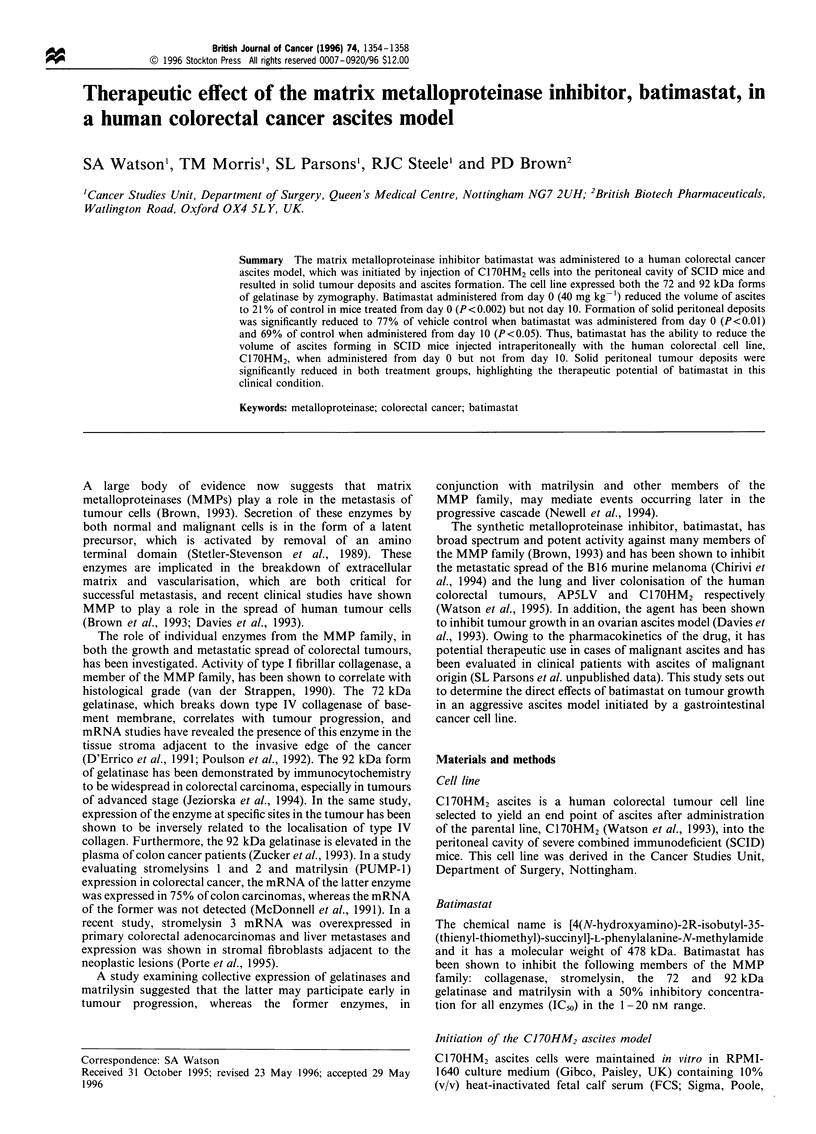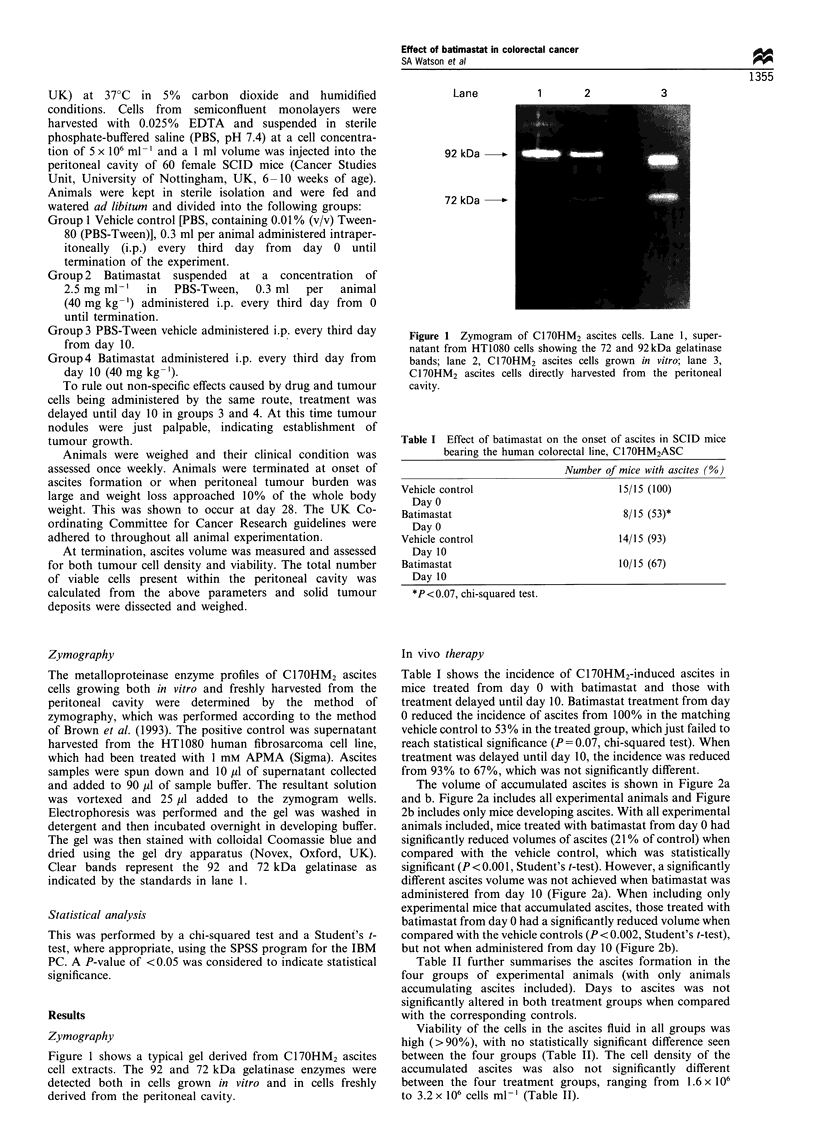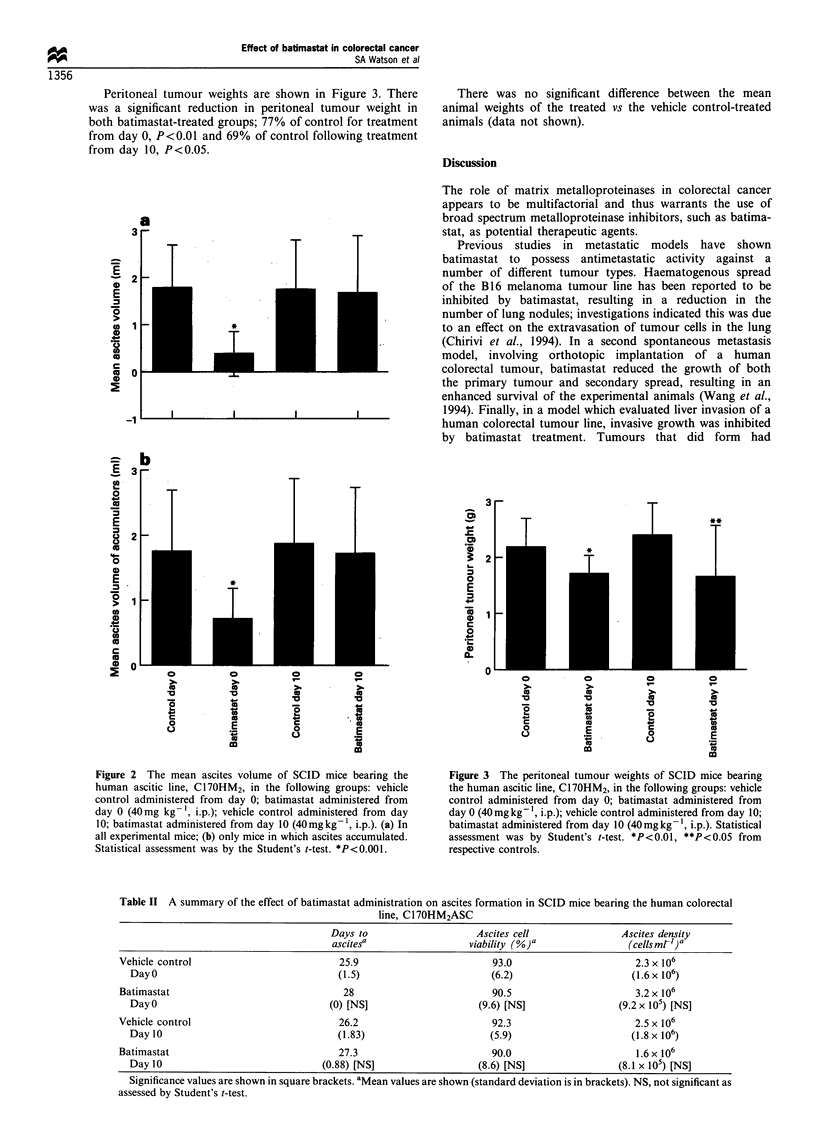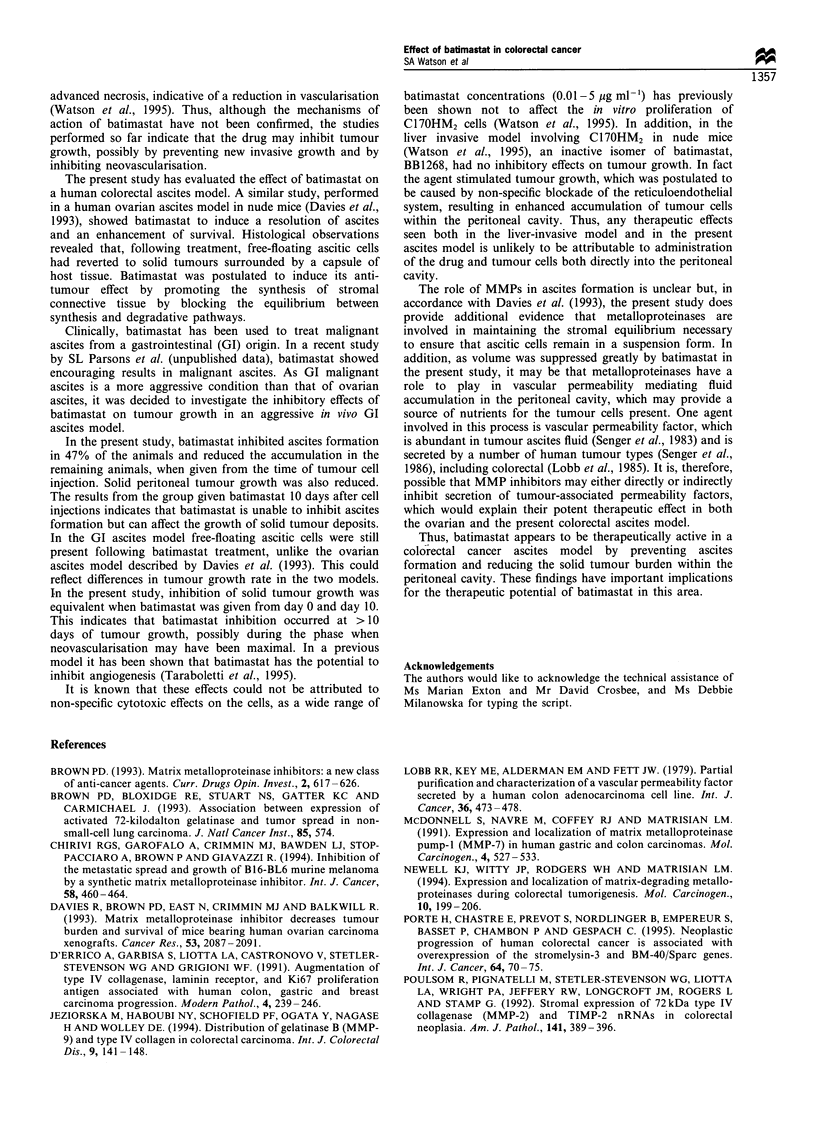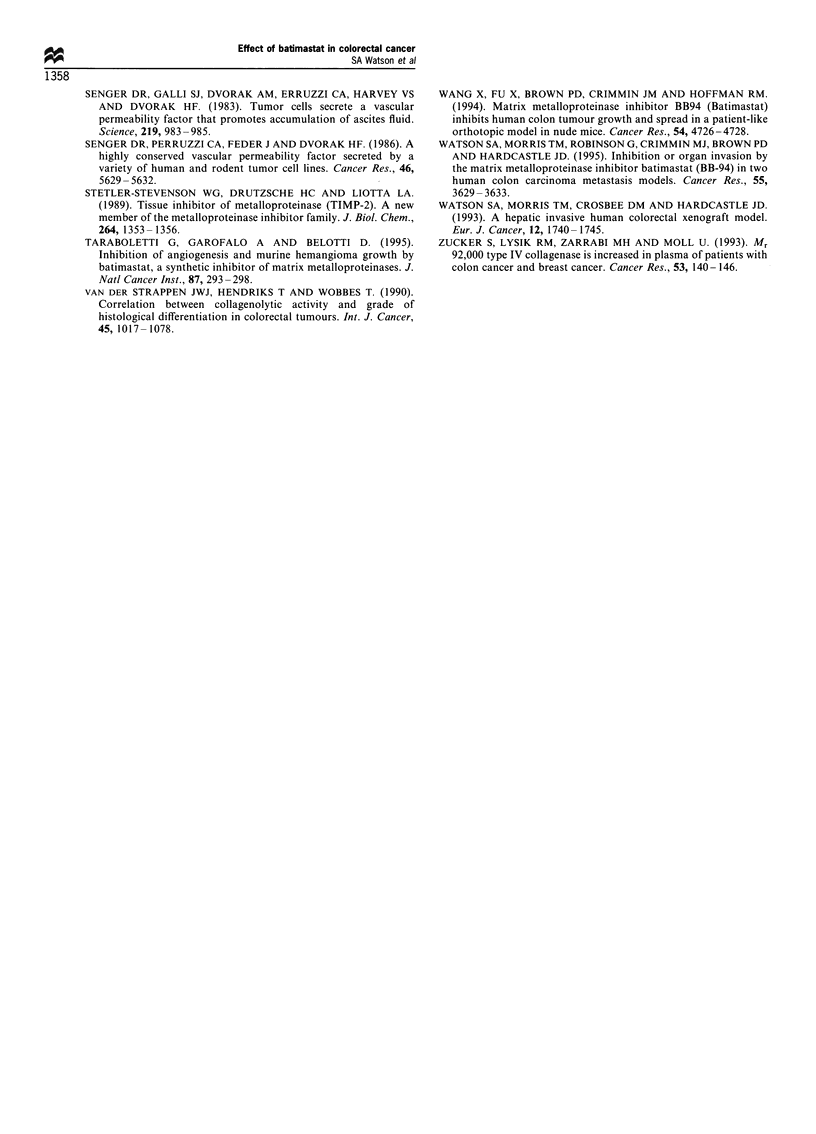# Therapeutic effect of the matrix metalloproteinase inhibitor, batimastat, in a human colorectal cancer ascites model.

**DOI:** 10.1038/bjc.1996.549

**Published:** 1996-11

**Authors:** S. A. Watson, T. M. Morris, S. L. Parsons, R. J. Steele, P. D. Brown

**Affiliations:** Department of Surgery, Queen's Medical Centre, Nottingham, UK.

## Abstract

**Images:**


British Journal of Cancer (1996) 74, 1354-1358
© 1996 Stockton Press All rights reserved 0007-0920/96 $12.00

### Therapeutic effect of the matrix metalloproteinase inhibitor, batimastat, in a human colorectal cancer ascites model

SA Watson[1], TM Morris[1], SL Parsons[1], RJC Steele[1] and PD Brown[2]

[1]*Cancer Studies Unit, Department of Surgery, Queen's Medical Centre, Nottingham NG7 2UH;* [2]*British Biotech Pharmaceuticals, Watlington Road, Oxford OX4 5LY, UK.*

**Summary** The matrix metalloproteinase inhibitor batimastat was administered to a human colorectal cancer ascites model, which was initiated by injection of C170HM$_2$ cells into the peritoneal cavity of SCID mice and resulted in solid tumour deposits and ascites formation. The cell line expressed both the 72 and 92 kDa forms of gelatinase by zymography. Batimastat administered from day 0 (40 mg kg$^{-1}$) reduced the volume of ascites to 21% of control in mice treated from day 0 ($P<0.002$) but not day 10. Formation of solid peritoneal deposits was significantly reduced to 77% of vehicle control when batimastat was administered from day 0 ($P<0.01$) and 69% of control when administered from day 10 ($P<0.05$). Thus, batimastat has the ability to reduce the volume of ascites forming in SCID mice injected intraperitoneally with the human colorectal cell line, C170HM$_2$, when administered from day 0 but not from day 10. Solid peritoneal tumour deposits were significantly reduced in both treatment groups, highlighting the therapeutic potential of batimastat in this clinical condition.

**Keywords:** metalloproteinase; colorectal cancer; batimastat

A large body of evidence now suggests that matrix metalloproteinases (MMPs) play a role in the metastasis of tumour cells (Brown, 1993). Secretion of these enzymes by both normal and malignant cells is in the form of a latent precursor, which is activated by removal of an amino terminal domain (Stetler-Stevenson *et al.*, 1989). These enzymes are implicated in the breakdown of extracellular matrix and vascularisation, which are both critical for successful metastasis, and recent clinical studies have shown MMP to play a role in the spread of human tumour cells (Brown *et al.*, 1993; Davies *et al.*, 1993).

The role of individual enzymes from the MMP family, in both the growth and metastatic spread of colorectal tumours, has been investigated. Activity of type I fibrillar collagenase, a member of the MMP family, has been shown to correlate with histological grade (van der Strappen, 1990). The 72 kDa gelatinase, which breaks down type IV collagenase of basement membrane, correlates with tumour progression, and mRNA studies have revealed the presence of this enzyme in the tissue stroma adjacent to the invasive edge of the cancer (D'Errico *et al.*, 1991; Poulson *et al.*, 1992). The 92 kDa form of gelatinase has been demonstrated by immunocytochemistry to be widespread in colorectal carcinoma, especially in tumours of advanced stage (Jeziorska *et al.*, 1994). In the same study, expression of the enzyme at specific sites in the tumour has been shown to be inversely related to the localisation of type IV collagen. Furthermore, the 92 kDa gelatinase is elevated in the plasma of colon cancer patients (Zucker *et al.*, 1993). In a study evaluating stromelysins 1 and 2 and matrilysin (PUMP-1) expression in colorectal cancer, the mRNA of the latter enzyme was expressed in 75% of colon carcinomas, whereas the mRNA of the former was not detected (McDonnell *et al.*, 1991). In a recent study, stromelysin 3 mRNA was overexpressed in primary colorectal adenocarcinomas and liver metastases and expression was shown in stromal fibroblasts adjacent to the neoplastic lesions (Porte *et al.*, 1995).

A study examining collective expression of gelatinases and matrilysin suggested that the latter may participate early in tumour progression, whereas the former enzymes, in conjunction with matrilysin and other members of the MMP family, may mediate events occurring later in the progressive cascade (Newell *et al.*, 1994).

The synthetic metalloproteinase inhibitor, batimastat, has broad spectrum and potent activity against many members of the MMP family (Brown, 1993) and has been shown to inhibit the metastatic spread of the B16 murine melanoma (Chirivi *et al.*, 1994) and the lung and liver colonisation of the human colorectal tumours, AP5LV and C170HM$_2$ respectively (Watson *et al.*, 1995). In addition, the agent has been shown to inhibit tumour growth in an ovarian ascites model (Davies *et al.*, 1993). Owing to the pharmacokinetics of the drug, it has potential therapeutic use in cases of malignant ascites and has been evaluated in clinical patients with ascites of malignant origin (SL Parsons *et al.* unpublished data). This study sets out to determine the direct effects of batimastat on tumour growth in an aggressive ascites model initiated by a gastrointestinal cancer cell line.

### Materials and methods

### *Cell line*

C170HM$_2$ ascites is a human colorectal tumour cell line selected to yield an end point of ascites after administration of the parental line, C170HM$_2$ (Watson *et al.*, 1993), into the peritoneal cavity of severe combined immunodeficient (SCID) mice. This cell line was derived in the Cancer Studies Unit, Department of Surgery, Nottingham.

### *Batimastat*

The chemical name is [4(*N*-hydroxyamino)-2R-isobutyl-35-(thienyl-thiomethyl)-succinyl]-L-phenylalanine-*N*-methylamide and it has a molecular weight of 478 kDa. Batimastat has been shown to inhibit the following members of the MMP family: collagenase, stromelysin, the 72 and 92 kDa gelatinase and matrilysin with a 50% inhibitory concentration for all enzymes (IC$_{50}$) in the 1–20 nM range.

### *Initiation of the C170HM$_2$ ascites model*

C170HM$_2$ ascites cells were maintained *in vitro* in RPMI-1640 culture medium (Gibco, Paisley, UK) containing 10% (v/v) heat-inactivated fetal calf serum (FCS; Sigma, Poole,

Correspondence: SA Watson
Received 31 October 1995; revised 23 May 1996; accepted 29 May 1996

UK) at 37°C in 5% carbon dioxide and humidified conditions. Cells from semiconfluent monolayers were harvested with 0.025% EDTA and suspended in sterile phosphate-buffered saline (PBS, pH 7.4) at a cell concentration of $5 \times 10^6$ ml$^{-1}$ and a 1 ml volume was injected into the peritoneal cavity of 60 female SCID mice (Cancer Studies Unit, University of Nottingham, UK, 6–10 weeks of age). Animals were kept in sterile isolation and were fed and watered *ad libitum* and divided into the following groups:

Group 1 Vehicle control [PBS, containing 0.01% (v/v) Tween-80 (PBS-Tween)], 0.3 ml per animal administered intraperitoneally (i.p.) every third day from day 0 until termination of the experiment.

Group 2 Batimastat suspended at a concentration of 2.5 mg ml$^{-1}$ in PBS-Tween, 0.3 ml per animal (40 mg kg$^{-1}$) administered i.p. every third day from 0 until termination.

Group 3 PBS-Tween vehicle administered i.p. every third day from day 10.

Group 4 Batimastat administered i.p. every third day from day 10 (40 mg kg$^{-1}$).

To rule out non-specific effects caused by drug and tumour cells being administered by the same route, treatment was delayed until day 10 in groups 3 and 4. At this time tumour nodules were just palpable, indicating establishment of tumour growth.

Animals were weighed and their clinical condition was assessed once weekly. Animals were terminated at onset of ascites formation or when peritoneal tumour burden was large and weight loss approached 10% of the whole body weight. This was shown to occur at day 28. The UK Co-ordinating Committee for Cancer Research guidelines were adhered to throughout all animal experimentation.

At termination, ascites volume was measured and assessed for both tumour cell density and viability. The total number of viable cells present within the peritoneal cavity was calculated from the above parameters and solid tumour deposits were dissected and weighed.

### *Zymography*

The metalloproteinase enzyme profiles of C170HM$_2$ ascites cells growing both *in vitro* and freshly harvested from the peritoneal cavity were determined by the method of zymography, which was performed according to the method of Brown *et al.* (1993). The positive control was supernatant harvested from the HT1080 human fibrosarcoma cell line, which had been treated with 1 mM APMA (Sigma). Ascites samples were spun down and 10 $\mu$l of supernatant collected and added to 90 $\mu$l of sample buffer. The resultant solution was vortexed and 25 $\mu$l added to the zymogram wells. Electrophoresis was performed and the gel was washed in detergent and then incubated overnight in developing buffer. The gel was then stained with colloidal Coomassie blue and dried using the gel dry apparatus (Novex, Oxford, UK). Clear bands represent the 92 and 72 kDa gelatinase as indicated by the standards in lane 1.

### *Statistical analysis*

This was performed by a chi-squared test and a Student's *t*-test, where appropriate, using the SPSS program for the IBM PC. A *P*-value of <0.05 was considered to indicate statistical significance.

### Results

### *Zymography*

Figure 1 shows a typical gel derived from C170HM$_2$ ascites cell extracts. The 92 and 72 kDa gelatinase enzymes were detected both in cells grown *in vitro* and in cells freshly derived from the peritoneal cavity.

**Figure 1** Zymogram of C170HM$_2$ ascites cells. Lane 1, supernatant from HT1080 cells showing the 72 and 92 kDa gelatinase bands; lane 2, C170HM$_2$ ascites cells grown *in vitro*; lane 3, C170HM$_2$ ascites cells directly harvested from the peritoneal cavity.

**Table I** Effect of batimastat on the onset of ascites in SCID mice bearing the human colorectal line, C170HM$_2$ASC

| | *Number of mice with ascites (%)* |
|---|---|
| Vehicle control Day 0 | 15/15 (100) |
| Batimastat Day 0 | 8/15 (53)* |
| Vehicle control Day 10 | 14/15 (93) |
| Batimastat Day 10 | 10/15 (67) |

*$P<0.07$, chi-squared test.

### In vivo *therapy*

Table I shows the incidence of C170HM$_2$-induced ascites in mice treated from day 0 with batimastat and those with treatment delayed until day 10. Batimastat treatment from day 0 reduced the incidence of ascites from 100% in the matching vehicle control to 53% in the treated group, which just failed to reach statistical significance ($P=0.07$, chi-squared test). When treatment was delayed until day 10, the incidence was reduced from 93% to 67%, which was not significantly different.

The volume of accumulated ascites is shown in Figure 2a and b. Figure 2a includes all experimental animals and Figure 2b includes only mice developing ascites. With all experimental animals included, mice treated with batimastat from day 0 had significantly reduced volumes of ascites (21% of control) when compared with the vehicle control, which was statistically significant ($P<0.001$, Student's *t*-test). However, a significantly different ascites volume was not achieved when batimastat was administered from day 10 (Figure 2a). When including only experimental mice that accumulated ascites, those treated with batimastat from day 0 had a significantly reduced volume when compared with the vehicle controls ($P<0.002$, Student's *t*-test), but not when administered from day 10 (Figure 2b).

Table II further summarises the ascites formation in the four groups of experimental animals (with only animals accumulating ascites included). Days to ascites was not significantly altered in both treatment groups when compared with the corresponding controls.

Viability of the cells in the ascites fluid in all groups was high (>90%), with no statistically significant difference seen between the four groups (Table II). The cell density of the accumulated ascites was also not significantly different between the four treatment groups, ranging from $1.6 \times 10^6$ to $3.2 \times 10^6$ cells ml$^{-1}$ (Table II).

Peritoneal tumour weights are shown in Figure 3. There was a significant reduction in peritoneal tumour weight in both batimastat-treated groups; 77% of control for treatment from day 0, $P<0.01$ and 69% of control following treatment from day 10, $P<0.05$.

There was no significant difference between the mean animal weights of the treated *vs* the vehicle control-treated animals (data not shown).

### Discussion

The role of matrix metalloproteinases in colorectal cancer appears to be multifactorial and thus warrants the use of broad spectrum metalloproteinase inhibitors, such as batimastat, as potential therapeutic agents.

Previous studies in metastatic models have shown batimastat to possess antimetastatic activity against a number of different tumour types. Haematogenous spread of the B16 melanoma tumour line has been reported to be inhibited by batimastat, resulting in a reduction in the number of lung nodules; investigations indicated this was due to an effect on the extravasation of tumour cells in the lung (Chirivi *et al.*, 1994). In a second spontaneous metastasis model, involving orthotopic implantation of a human colorectal tumour, batimastat reduced the growth of both the primary tumour and secondary spread, resulting in an enhanced survival of the experimental animals (Wang *et al.*, 1994). Finally, in a model which evaluated liver invasion of a human colorectal tumour line, invasive growth was inhibited by batimastat treatment. Tumours that did form had

**Figure 2** The mean ascites volume of SCID mice bearing the human ascitic line, C170HM$_2$, in the following groups: vehicle control administered from day 0; batimastat administered from day 0 (40 mg kg$^{-1}$, i.p.); vehicle control administered from day 10; batimastat administered from day 10 (40 mg kg$^{-1}$, i.p.). (**a**) In all experimental mice; (**b**) only mice in which ascites accumulated. Statistical assessment was by the Student's *t*-test. $^*P<0.001$.

**Figure 3** The peritoneal tumour weights of SCID mice bearing the human ascitic line, C170HM$_2$, in the following groups: vehicle control administered from day 0; batimastat administered from day 0 (40 mg kg$^{-1}$, i.p.); vehicle control administered from day 10; batimastat administered from day 10 (40 mg kg$^{-1}$, i.p.). Statistical assessment was by Student's *t*-test. $^*P<0.01$, $^{**}P<0.05$ from respective controls.

**Table II** A summary of the effect of batimastat administration on ascites formation in SCID mice bearing the human colorectal line, C170HM$_2$ASC

| | *Days to ascites*[a] | *Ascites cell viability (%)*[a] | *Ascites density (cells ml$^{-1}$)*[a] |
|---|---|---|---|
| Vehicle control Day 0 | 25.9 (1.5) | 93.0 (6.2) | $2.3\times10^6$ ($1.6\times10^6$) |
| Batimastat Day 0 | 28 (0) [NS] | 90.5 (9.6) [NS] | $3.2\times10^6$ ($9.2\times10^5$) [NS] |
| Vehicle control Day 10 | 26.2 (1.83) | 92.3 (5.9) | $2.5\times10^6$ ($1.8\times10^6$) |
| Batimastat Day 10 | 27.3 (0.88) [NS] | 90.0 (8.6) [NS] | $1.6\times10^6$ ($8.1\times10^5$) [NS] |

Significance values are shown in square brackets. [a]Mean values are shown (standard deviation is in brackets). NS, not significant as assessed by Student's *t*-test.

advanced necrosis, indicative of a reduction in vascularisation (Watson *et al.*, 1995). Thus, although the mechanisms of action of batimastat have not been confirmed, the studies performed so far indicate that the drug may inhibit tumour growth, possibly by preventing new invasive growth and by inhibiting neovascularisation.

The present study has evaluated the effect of batimastat on a human colorectal ascites model. A similar study, performed in a human ovarian ascites model in nude mice (Davies *et al.*, 1993), showed batimastat to induce a resolution of ascites and an enhancement of survival. Histological observations revealed that, following treatment, free-floating ascitic cells had reverted to solid tumours surrounded by a capsule of host tissue. Batimastat was postulated to induce its anti-tumour effect by promoting the synthesis of stromal connective tissue by blocking the equilibrium between synthesis and degradative pathways.

Clinically, batimastat has been used to treat malignant ascites from a gastrointestinal (GI) origin. In a recent study by SL Parsons *et al.* (unpublished data), batimastat showed encouraging results in malignant ascites. As GI malignant ascites is a more aggressive condition than that of ovarian ascites, it was decided to investigate the inhibitory effects of batimastat on tumour growth in an aggressive *in vivo* GI ascites model.

In the present study, batimastat inhibited ascites formation in 47% of the animals and reduced the accumulation in the remaining animals, when given from the time of tumour cell injection. Solid peritoneal tumour growth was also reduced. The results from the group given batimastat 10 days after cell injections indicates that batimastat is unable to inhibit ascites formation but can affect the growth of solid tumour deposits. In the GI ascites model free-floating ascitic cells were still present following batimastat treatment, unlike the ovarian ascites model described by Davies *et al.* (1993). This could reflect differences in tumour growth rate in the two models. In the present study, inhibition of solid tumour growth was equivalent when batimastat was given from day 0 and day 10. This indicates that batimastat inhibition occurred at >10 days of tumour growth, possibly during the phase when neovascularisation may have been maximal. In a previous model it has been shown that batimastat has the potential to inhibit angiogenesis (Taraboletti *et al.*, 1995).

It is known that these effects could not be attributed to non-specific cytotoxic effects on the cells, as a wide range of batimastat concentrations (0.01–5 $\mu g\ ml^{-1}$) has previously been shown not to affect the *in vitro* proliferation of $C170HM_2$ cells (Watson *et al.*, 1995). In addition, in the liver invasive model involving $C170HM_2$ in nude mice (Watson *et al.*, 1995), an inactive isomer of batimastat, BB1268, had no inhibitory effects on tumour growth. In fact the agent stimulated tumour growth, which was postulated to be caused by non-specific blockade of the reticuloendothelial system, resulting in enhanced accumulation of tumour cells within the peritoneal cavity. Thus, any therapeutic effects seen both in the liver-invasive model and in the present ascites model is unlikely to be attributable to administration of the drug and tumour cells both directly into the peritoneal cavity.

The role of MMPs in ascites formation is unclear but, in accordance with Davies *et al.* (1993), the present study does provide additional evidence that metalloproteinases are involved in maintaining the stromal equilibrium necessary to ensure that ascitic cells remain in a suspension form. In addition, as volume was suppressed greatly by batimastat in the present study, it may be that metalloproteinases have a role to play in vascular permeability mediating fluid accumulation in the peritoneal cavity, which may provide a source of nutrients for the tumour cells present. One agent involved in this process is vascular permeability factor, which is abundant in tumour ascites fluid (Senger *et al.*, 1983) and is secreted by a number of human tumour types (Senger *et al.*, 1986), including colorectal (Lobb *et al.*, 1985). It is, therefore, possible that MMP inhibitors may either directly or indirectly inhibit secretion of tumour-associated permeability factors, which would explain their potent therapeutic effect in both the ovarian and the present colorectal ascites model.

Thus, batimastat appears to be therapeutically active in a colorectal cancer ascites model by preventing ascites formation and reducing the solid tumour burden within the peritoneal cavity. These findings have important implications for the therapeutic potential of batimastat in this area.

**Acknowledgements**
The authors would like to acknowledge the technical assistance of Ms Marian Exton and Mr David Crosbee, and Ms Debbie Milanowska for typing the script.

### References

BROWN PD. (1993). Matrix metalloproteinase inhibitors: a new class of anti-cancer agents. *Curr. Drugs Opin. Invest.*, **2**, 617–626.

BROWN PD, BLOXIDGE RE, STUART NS, GATTER KC AND CARMICHAEL J. (1993). Association between expression of activated 72-kilodalton gelatinase and tumor spread in non-small-cell lung carcinoma. *J. Natl Cancer Inst.*, **85**, 574.

CHIRIVI RGS, GAROFALO A, CRIMMIN MJ, BAWDEN LJ, STOPPACCIARO A, BROWN P AND GIAVAZZI R. (1994). Inhibition of the metastatic spread and growth of B16-BL6 murine melanoma by a synthetic matrix metalloproteinase inhibitor. *Int. J. Cancer*, **58**, 460–464.

DAVIES R, BROWN PD, EAST N, CRIMMIN MJ AND BALKWILL R. (1993). Matrix metalloproteinase inhibitor decreases tumour burden and survival of mice bearing human ovarian carcinoma xenografts. *Cancer Res.*, **53**, 2087–2091.

D'ERRICO A, GARBISA S, LIOTTA LA, CASTRONOVO V, STETLER-STEVENSON WG AND GRIGIONI WF. (1991). Augmentation of type IV collagenase, laminin receptor, and Ki67 proliferation antigen associated with human colon, gastric and breast carcinoma progression. *Modern Pathol.*, **4**, 239–246.

JEZIORSKA M, HABOUBI NY, SCHOFIELD PF, OGATA Y, NAGASE H AND WOLLEY DE. (1994). Distribution of gelatinase B (MMP-9) and type IV collagen in colorectal carcinoma. *Int. J. Colorectal Dis.*, **9**, 141–148.

LOBB RR, KEY ME, ALDERMAN EM AND FETT JW. (1979). Partial purification and characterization of a vascular permeability factor secreted by a human colon adenocarcinoma cell line. *Int. J. Cancer*, **36**, 473–478.

McDONNELL S, NAVRE M, COFFEY RJ AND MATRISIAN LM. (1991). Expression and localization of matrix metalloproteinase pump-1 (MMP-7) in human gastric and colon carcinomas. *Mol. Carcinogen.*, **4**, 527–533.

NEWELL KJ, WITTY JP, RODGERS WH AND MATRISIAN LM. (1994). Expression and localization of matrix-degrading metalloproteinases during colorectal tumorigenesis. *Mol. Carcinogen.*, **10**, 199–206.

PORTE H, CHASTRE E, PREVOT S, NORDLINGER B, EMPEREUR S, BASSET P, CHAMBON P AND GESPACH C. (1995). Neoplastic progression of human colorectal cancer is associated with overexpression of the stromelysin-3 and BM-40/Sparc genes. *Int. J. Cancer*, **64**, 70–75.

POULSOM R, PIGNATELLI M, STETLER-STEVENSON WG, LIOTTA LA, WRIGHT PA, JEFFERY RW, LONGCROFT JM, ROGERS L AND STAMP G. (1992). Stromal expression of 72 kDa type IV collagenase (MMP-2) and TIMP-2 nRNAs in colorectal neoplasia. *Am. J. Pathol.*, **141**, 389–396.

SENGER DR, GALLI SJ, DVORAK AM, ERRUZZI CA, HARVEY VS AND DVORAK HF. (1983). Tumor cells secrete a vascular permeability factor that promotes accumulation of ascites fluid. *Science*, **219,** 983–985.

SENGER DR, PERRUZZI CA, FEDER J AND DVORAK HF. (1986). A highly conserved vascular permeability factor secreted by a variety of human and rodent tumor cell lines. *Cancer Res.*, **46,** 5629–5632.

STETLER-STEVENSON WG, DRUTZSCHE HC AND LIOTTA LA. (1989). Tissue inhibitor of metalloproteinase (TIMP-2). A new member of the metalloproteinase inhibitor family. *J. Biol. Chem.*, **264,** 1353–1356.

TARABOLETTI G, GAROFALO A AND BELOTTI D. (1995). Inhibition of angiogenesis and murine hemangioma growth by batimastat, a synthetic inhibitor of matrix metalloproteinases. *J. Natl Cancer Inst.*, **87,** 293–298.

VAN DER STRAPPEN JWJ, HENDRIKS T AND WOBBES T. (1990). Correlation between collagenolytic activity and grade of histological differentiation in colorectal tumours. *Int. J. Cancer*, **45,** 1017–1078.

WANG X, FU X, BROWN PD, CRIMMIN JM AND HOFFMAN RM. (1994). Matrix metalloproteinase inhibitor BB94 (Batimastat) inhibits human colon tumour growth and spread in a patient-like orthotopic model in nude mice. *Cancer Res.*, **54,** 4726–4728.

WATSON SA, MORRIS TM, ROBINSON G, CRIMMIN MJ, BROWN PD AND HARDCASTLE JD. (1995). Inhibition or organ invasion by the matrix metalloproteinase inhibitor batimastat (BB-94) in two human colon carcinoma metastasis models. *Cancer Res.*, **55,** 3629–3633.

WATSON SA, MORRIS TM, CROSBEE DM AND HARDCASTLE JD. (1993). A hepatic invasive human colorectal xenograft model. *Eur. J. Cancer*, **12,** 1740–1745.

ZUCKER S, LYSIK RM, ZARRABI MH AND MOLL U. (1993). $M_r$ 92,000 type IV collagenase is increased in plasma of patients with colon cancer and breast cancer. *Cancer Res.*, **53,** 140–146.